# Urinary monocyte chemotactic protein-1 (MCP-1) in leprosy patients: increased risk for kidney damage

**DOI:** 10.1186/1471-2334-14-451

**Published:** 2014-08-20

**Authors:** Gdayllon Cavalcante Meneses, Alexandre Braga Libório, Elizabeth Francesco de Daher, Geraldo Bezerra da Silva, Marcus Felipe Bezerra da Costa, Maria Araci Andrade Pontes, Alice Maria Costa Martins

**Affiliations:** Department of Physiology and Pharmacology, Faculty of Medicine, Federal University of Ceara, Fortaleza, Ceara Brazil; Department of Clinical Medicine, Faculty of Medicine, Federal University of Ceará, Fortaleza, Ceara Brazil; Hospital Dona Libânia, Fortaleza, Ceara Brazil; Department of Clinical and Toxicological Analysis, Faculty of Pharmacy, Federal University of Ceara, Fortaleza, Ceara Brazil; Post-Graduation Program in Collective Health, University of Fortaleza, Fortaleza, Ceara Brazil

## Abstract

**Background:**

We aimed to evaluate urinary MCP-1 and oxidative stress through urinary malondialdehyde (MDA) in leprosy and correlate them with traditional, but less sensitive markers of renal disease.

**Methods:**

This is a cross-sectional study of 44 patients with diagnosis of leprosy and no previous treatment. Skin smear was assessed through a bacteriological index - from 0 to 6+. Glomerular filtration rate (GFR), protein excretion rate, microalbuminuria, urinary oxidative stress, malondialdehyde (MDA) and urinary MCP-1 were measured. Also, high- sensitivity C-reactive protein (hs-CRP) was measured in the blood. Fifteen healthy subjects composed a control group.

**Results:**

Age and gender were similar between leprosy patients and control groups. No patient had a GFR < 60 mL/min/1.73 m2 or albumin excretion rate greater than 30 mg/g-Cr. Leprosy patients had higher urinary protein excretion (97.6 ± 69.2 vs. 6.5 ± 4.3 mg/g-Cr, p < 0.001), urinary MCP-1 (101.0 ± 79.8 vs. 34.5 ± 14.9 mg/g-Cr, p = 0.006) and urinary MDA levels (1.77 ± 1.31 vs. 1.27 ± 0.66 mmol/g-Cr, p = 0.0372) than healthy controls. There was a positive correlation between urinary MCP-1 and bacteriological index in skin smears (r = 0.322, p = 0.035), urinary protein excretion (r = 0.547, p < 0.001), albumin excretion rate (r = 0.414, p = 0.006) and urinary MDA (r = 0.453, p = 0.002). After adjusting for hs-CRP, urinary MCP-1 remained correlated with albumin excretion rate (r_partial_ = 0.483, p = 0.007) and MDA levels (r_partial_ = 0.555, p = 0.001).

**Conclusion:**

Leprosy patients with no clinical kidney disease have increased urinary MCP-1 mainly in lepromatous polar form. Inflammatory (MCP-1) and oxidative stress markers suggest leprosy patients are at high risk of developing kidney disease.

**Electronic supplementary material:**

The online version of this article (doi:10.1186/1471-2334-14-451) contains supplementary material, which is available to authorized users.

## Background

Leprosy, a chronic infectious disease caused by the obligate intracellular bacterium *Mycobacterium leprae*, remains a major source of morbidity in developing countries. The disease affects mainly the skin and peripheral nervous system, but it has a wide range of clinical and histopathological manifestations. Depending on the degree and efficacy of cell-mediated immunity, patients can present with a single, well-demarcated lesion - polar tuberculoid (TT) or, on the other extreme, with numerous, poorly demarcated, raised or nodular lesions on all parts of the body, which constitutes polar lepromatous (LL) leprosy. Other patients, however, fall into a broad borderline category between these two polar forms; this is subdivided into borderline lepromatous (BL), mid-borderline (BB), and borderline tuberculoid (BT) [1, 2].

Renal lesions in leprosy patients can be present in up to 72% of leprosy patients in autopsied patients [3]; however, clinical manifestation can be subtle. Only a few patients show a fast decline in renal function or major proteinuria associated with edema. These uncommon cases are published as case reports, due to their rarity [4, 5]. Many other leprosy patients can be at risk for developing kidney disease. Identification of these patients is difficult in part due to lack of sensitivity of diagnostic tests used in clinical practice to detect incipient renal disease.

The monocyte chemotactic protein-1 (MCP-1) is one of the new recently studied renal biomarkers. This protein is expressed in injury and inflammation sites and directs the recruitment of macrophages, which bind to chemokine receptors to promote macrophage adhesion and chemotaxis. The renal increase in MCP-1 expression can occur in progressive kidney disease and when there is interstitial inflammatory infiltrate [6]. Urinary MCP-1 has been associated with increased albuminuria in patients with other renal diseases, such as diabetes [7]. Also, investigations suggest that oxidative stress plays a pivotal role in the pathogenesis, progression, and complications of kidney disease [8]. Several urinary biomarkers of oxidative stress have been studied in the context of progressive kidney disease [9, 10].

In the present study, we aimed to evaluate urinary MCP-1 and oxidative stress through urinary malondialdehyde (MDA) in leprosy patients in comparison with a healthy control group and correlate them with traditional, but less sensitive markers of renal disease. In addition, we compared patients according to bacilli smear positive cases and polar leprosy clinical picture.

## Methods

This is a cross-sectional study of 44 patients with clinical and laboratory diagnosis of leprosy. Data were collected among patients diagnosed with leprosy with no previous anti-mycobacterium treatment. Patients were recruited in public health centers from Fortaleza, state of Ceara, Brazil between August 2012 and August 2013. This study protocol was approved by the Ethical Committee of Walter Cantidio University Hospital, Universidade Federal do Ceara, Brazil (N. 267.426) and all participants signed informed consent. Exclusion criteria included: patients with known previous renal disease (according to Kidney Disease Outcomes Quality Initiative recommendations - glomerular filtration rate higher than 60 mL/min/1.73 m [2] and albumin excretion rate less than 30 mg/g-Cr), diabetes mellitus, systemic lupus erythematosus, arterial hypertension and erythema nodosum leprosum reaction episode. Also, a group of 15 healthy subjects were included as a control group.

The following data were collected: age, gender, time of symptoms, use of other concomitant drugs, number of skin lesions, skin smear microscopy, systolic and diastolic blood pressure. Leprosy was classified clinically as tuberculoid polar form (TT and BT), mid-borderline (BB) and lepromatous polar form (BL and LL). Also, patients were classified regarding smear-positivity as paucibacillary (no bacilli smear-positive) or multibacillary. Skin smear was assessed through a bacteriological index - from 0 to 6+ [2]. Serum creatinine and high-sensitivity C-reactive protein (hs-CRP) were measured. Additionally, a morning urine sample (after approximately an 8-hour fasting period) was collected and creatinine, total protein, microalbuminuria, MDA and MCP-1 levels were measured. All urine measurements were normalized by urinary creatinine concentration. Glomerular filtration rate (GFR) was estimated using CKD-EPI equation.

The analytical methods used for creatinine and urine protein excretion, measurement were colorimetric methods (Labtest®). C-reactive protein (CRP) and microalbuminuria were quantified by automated immunoturbidimetry (Cobas C 111, Roche®). Urinary malondialdehyde (MDA) was isolated and quantified using the thiobarbituric acid (TBARS) test. Urinary MCP-1 was determined by sandwich enzyme-linked immunosorbent assay (ELISA) (Boster Biological Technology, Fremont, CA, USA).

Data were tested for normal distribution and are shown as mean ± S.D. Groups were compared using t-student test or one-way-ANOVA with Bonferroni post-test. Correlations were performed using Pearson’s coefficient. A p-value less than 0.05 was considered significant.

## Results

**Figure 1:**
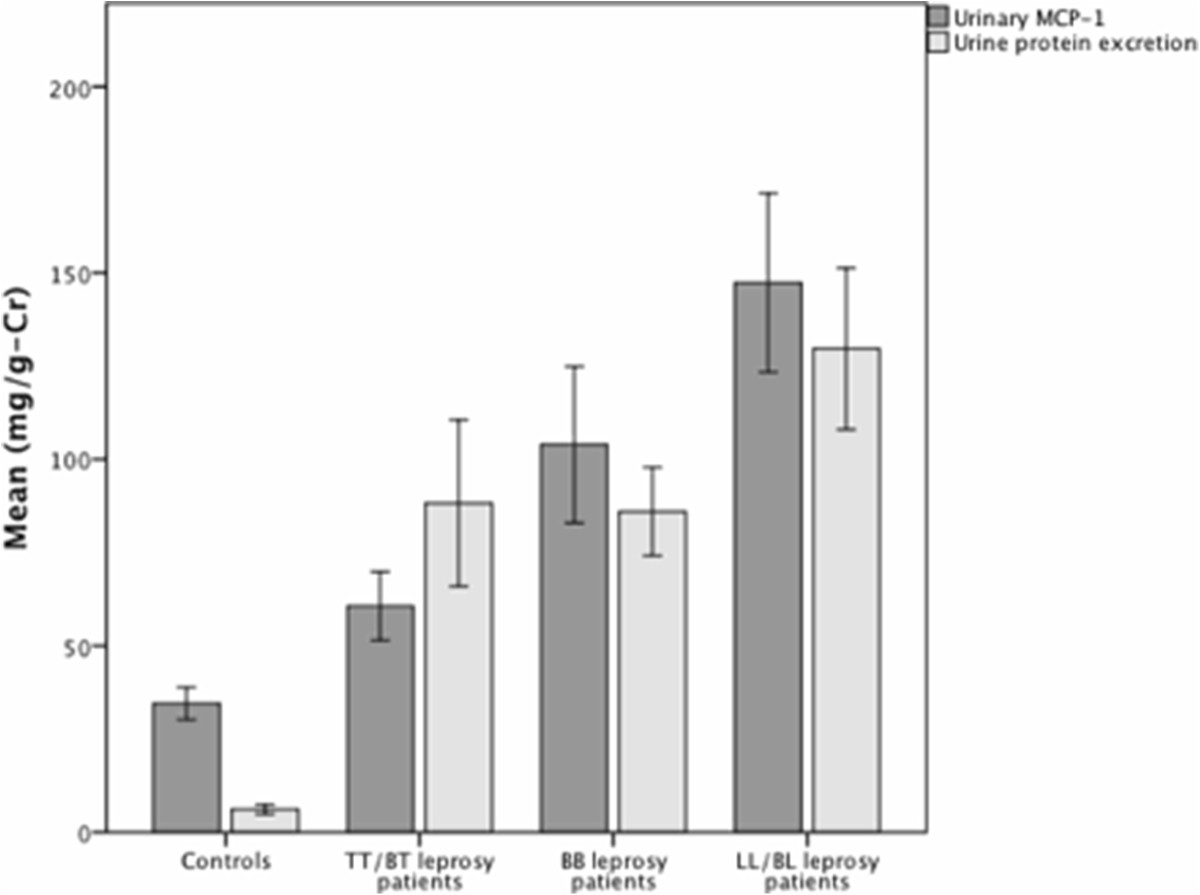
**Urinary MCP-1 and protein excretion in controls and leprosy patients according their clinical classification.**

A total of 44 patients (63.6% males) with a mean age of 36.1 ± 10.6 years old were selected. Additionally, 15 healthy patients were selected as controls - mean age 35.4 ± 9.2. Time from symptom onset to leprosy diagnosis ranged from one month to 8 years, with a median time of 17 months. Twenty-six patients had positive skin-smear test (multibacillary – MB) and 18 were paucibacillary - PB. Clinically, there were 14 TT/BT, 19 BB and 11 LL/BL leprosy patients. Regarding renal function, no patient had chronic kidney disease according to Kidney Disease Outcomes Quality Initiative definition - glomerular filtration rate higher than 60 mL/min/1.73 m [2] and albumin excretion rate less than 30 mg/g-Cr.Leprosy patients had higher urinary MCP-1 (101.0 ± 79.8 vs. 34.5 ± 14.9 mg/g-Cr, p = 0.006) and urinary MDA levels (1.77 ± 1.31 vs. 1.27 ± 0.66 mmol/g-Cr, p = 0.0372) than healthy controls. Also, urinary protein excretion was higher in leprosy patients when compared to controls (97.6 ± 69.2 vs. 6.5 ± 4.3 mg/g-Cr, p < 0.001). Moreover, there was a stepwise increment in urinary MCP-1 and urine protein excretion rate values from tuberculoid to lepromatous type in leprosy patients – see Figure 1. Urinary MCP-1 was higher in multibacillary than in paucibacillary patients (122.1 ± 91.9 vs. 72.0 ± 46.1 mg/g-Cr, p = 0.023) and there was a positive correlation between urinary MCP-1 and bacteriological index in skin smears (r = 0.322, p = 0.035). No significant correlation between urinary MCP-1 and time of symptoms was observed (r = 0.014, p = 0.938).

**Figure 2:**
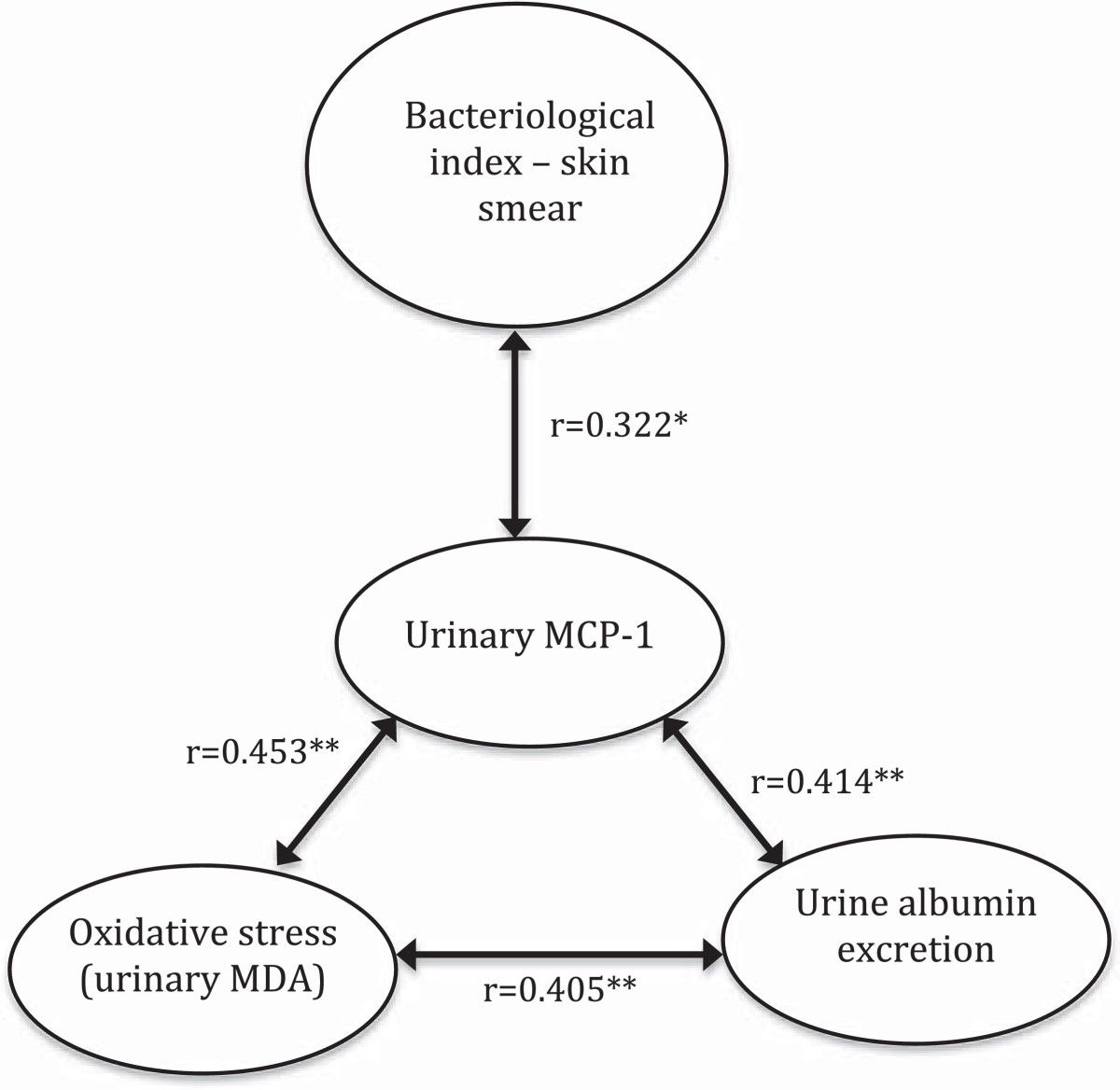
**Pearson correlations between number bacteriological index in skin smear, urinary monocyte chemotactic protein-1 (MCP-1), urinary malondialdehyde and urine albumin excretion.** *p < 0.05, **p < 0.01.

Although there was no correlation between glomerular filtration rate and urinary MCP-1 levels (r = -0.018, p = 0.906), urinary MCP-1 was correlated with urinary protein excretion (r = 0.547, p < 0.001), albumin excretion rate (r = 0.414, p = 0.006) and urinary MDA (r = 0.453, p = 0.002) – Figure 2. To exclude the possibility that elevated urinary MCP-1 would be only a reflex of systemic inflammation, we also measured serum hs-CRP and there was no correlation between both markers (r = -0.095, p = 0.606). After adjusting for hs-CRP, urinary MCP-1 remained correlated with albumin excretion rate (r_partial_ = 0.483, p = 0.007) and MDA levels (r_partial_ = 0.555, p = 0.001) – Table 1.

**Table 1:** **Pearson and partial correlation between MCP**-**1 and others parameters of renal function**/**biomarkers**

	Pearson correlation	P	Partial correlation (adjusted for hs-CRP)	p
Glomerular fitration rate	-0.018	0.906	0.002	0.934
Urinary protein excretion	0.547	<0.001	0.526	0.002
Albumin excretion rate	0.414	0.006	0.483	0.007
Urinary MDA	0.453	0.002	0.555	0.001

*Hs*-*CRP*: high-sensibility C-reactive protein; MDA: malondialdehyde.

## Discussion

In the present study, leprosy patients with no clinical kidney disease had increased urinary MCP-1 and MDA, new markers of kidney disease progression. Moreover, patients with multibacillary and lepromatous polar form of leprosy had increased levels of urinary MCP-1. Albumin excretion rate, a known marker of kidney disease progression, correlated with urinary MCP-1. Also, there was a positive correlation between urinary MCP-1 and MDA.

Leprosy patients can present with kidney disease of glomerular (glomerulonephritis/amyloidosis) or tubule-interstitial etiology. Generally, these patients present clinically with overt proteinuria or reduced GFR. Although they have been well described, these pathologies are not common and are generally published as case reports. However, leprosy patients have an increased risk of developing chronic kidney disease and subclinical renal lesions can be a common event [11]. In this study, leprosy patients with no clinical kidney disease showed increased levels of urinary MCP-1. This is a relatively new biomarker that has been associated with kidney disease progression in other pathologies, such as diabetic nephropathy. Although patients with active lupus nephritis [12] had higher urinary MCP-1 levels than leprosy patients; these had urinary MCP-1 level comparable to diabetic patients with microalbuminuria [13].

Interestingly, urinary MCP-1 levels were correlated with the lepromatous polar form and the bacteriological index in skin smears. This correlation is probably due to increased immunocomplexes in renal disease in multibacillary patients, resulting in higher inflammation burden. However, we cannot exclude the possibility that a predominant T_h_2 immune response can be responsible for renal inflammation in these patients. Monocyte chemoattractant protein–1 has been recognized as a crucial factor for the development of adaptive T_h_2 responses [14], making the second hypothesis plausible.

Urinary oxidative stress was increased in leprosy patients and correlated with urinary MCP-1 levels. Recently, kidney MCP-1 expression was correlated with oxidative stress in diabetic nephropathy [15]. According to Figure 2, we suggest that increased MCP-1 levels increase renal oxidative stress, contributing to renal damage.

Both urinary MCP-1 and oxidative stress correlated with urinary protein and albumin excretion rate. Urinary albumin excretion rate is a known marker of kidney disease progression and studies have indicated a continuous risk increment with high levels of urinary albumin excretion, even when it remains within the normal range [16]. This fact indicates that leprosy patients can be at risk of developing advanced renal disease in the future. Considering that more than 15% of leprosy patients can show elevated urine albumin excretion [17], urinary MCP-1 can be a useful early biomarker to identify patients at risk. Although, leprosy is not a recognized cause of clinical kidney disease, it may be due to the long time to developing chronic kidney damage. It is probable that a long-term follow-up in leprosy patients can detect a higher incidence of chronic kidney disease in comparison with normal population.

The possibility that urinary MCP-1 was only a manifestation of systemic inflammation was considered, but even after adjusting for hs-CRP, urinary MCP-1 remained correlated with urine albumin and protein excretion rates, indicating that renal MCP-1 expression is increased in these patients, regardless of systemic inflammation.

The main limitation of the present study is its cross-sectional design. Because of its design, we cannot affirm there is a causal relationship between urinary MCP-1, oxidative stress and urinary albumin excretion. Also, cohort prospective studies are necessary to determine whether these subclinical renal alterations will develop into the clinically manifest disease.

## Conclusion

We demonstrated that leprosy patients with no clinical kidney disease have increased urinary MCP-1 and its levels are even higher in patients with the lepromatous polar form. Moreover, urinary MCP-1 was associated with urinary oxidative stress and urinary albumin excretion, suggesting these patients are at increased risk of developing clinical kidney disease.

## Authors’ original submitted files for images

Below are the links to the authors’ original submitted files for images. Authors’ original file for figure 1 Authors’ original file for figure 2
