# Supplementary figures and images for: Urinary monocyte chemotactic protein-1 (MCP-1) in leprosy patients: increased risk for kidney damage
Source: BMC Infect Dis. 2014 Aug 20;14:451. doi: 10.1186/1471-2334-14-451 (PMC4158081; doi:10.1186/1471-2334-14-451)

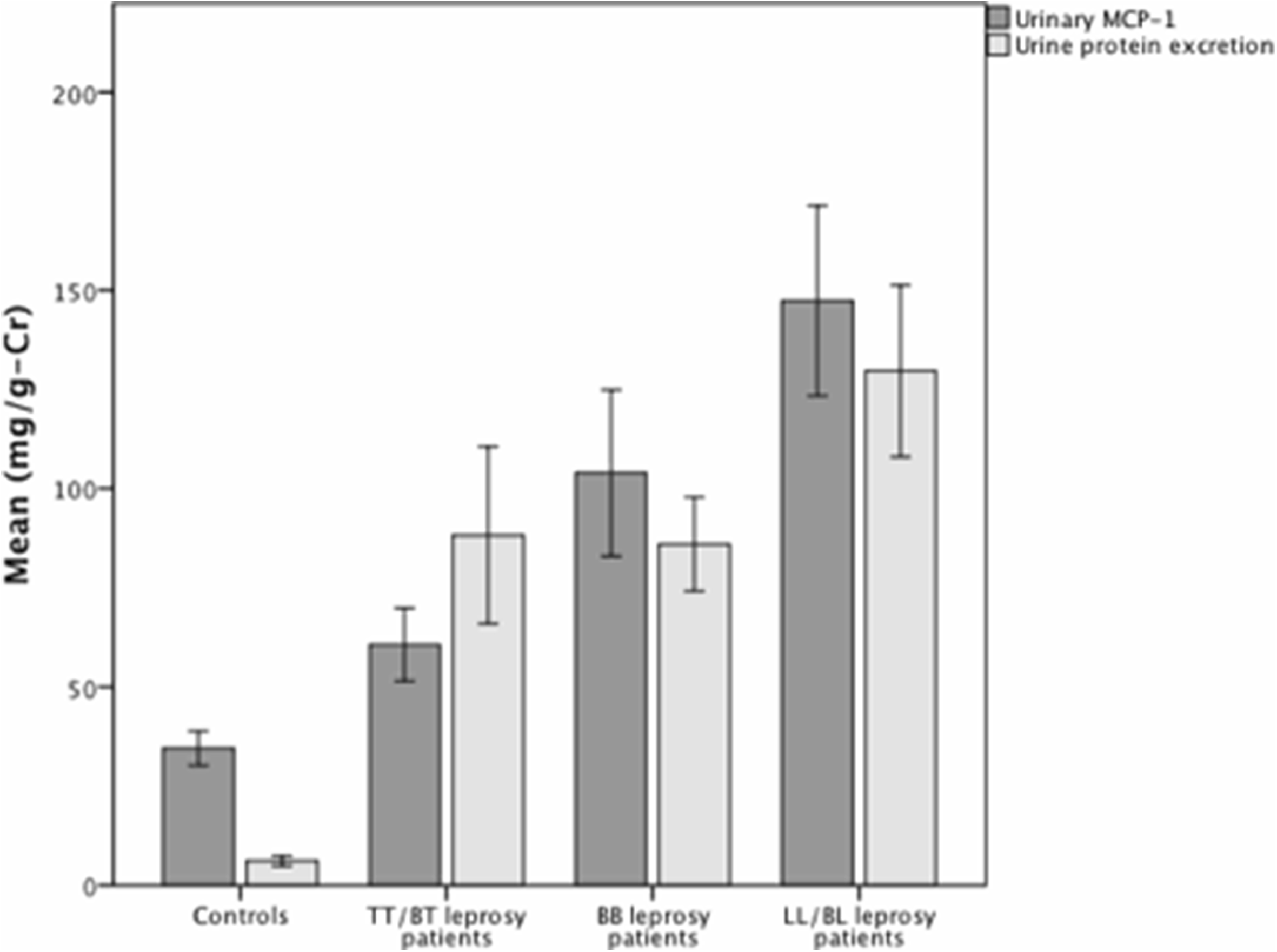

Supplement: Supplementary file 1 — Authors’ original file for figure 1 [file 12879_2014_3777_MOESM1_ESM.tif]

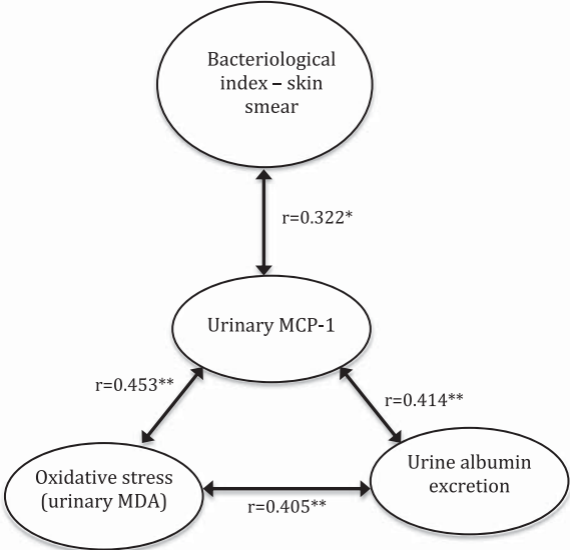

Supplement: Supplementary file 2 — Authors’ original file for figure 2 [file 12879_2014_3777_MOESM2_ESM.pdf]
